# Effect of Intraoperative Goal-Directed Fluid Management on Tissue Oxygen Tension in Obese Patients: a Randomized Controlled Trial

**DOI:** 10.1007/s11695-020-05106-x

**Published:** 2020-11-27

**Authors:** Jakob Mühlbacher, Florian Luf, Oliver Zotti, Harald Herkner, Edith Fleischmann, Barbara Kabon

**Affiliations:** 1 Department of Surgery, Division of General Surgery, Medical University of Vienna, Spitalgasse 23, 1090 Vienna, Austria; 2 Department of Anaesthesiology and Intensive Care, Hanusch Hospital, Heinrich-Collin-Strasse 30, 1140 Vienna, Austria; 3 Department of Emergency Medicine, Medical University of Vienna, Waehringer Guertel 18-20, 1090 Vienna, Austria; 4 Department of Anaesthesia, General Intensive Care and Pain Medicine, Medical University Vienna, Spitalgasse 23, A-1090 Vienna, Austria

**Keywords:** Obesity, Laparoscopic surgery, Goal-directed fluid therapy, Tissue oxygen tension

## Abstract

**Background:**

Perioperative subcutaneous tissue oxygen tension (PsqO_2_) is substantially reduced in obese surgical patients. Goal-directed fluid therapy optimizes cardiac performance and thus tissue perfusion and oxygen delivery. We therefore tested the hypothesis that intra- and postoperative PsqO_2_ is significantly reduced in obese patients undergoing standard fluid management compared to goal-directed fluid administration.

**Methods:**

We randomly assigned 60 obese patients (BMI ≥ 30 kg/m^2^) undergoing laparoscopic bariatric surgery to receive either esophageal Doppler-guided goal-directed fluid management or conventional fluid treatment. Our primary outcome parameter was intra- and postoperative PsqO_2_ measured with a polarographic electrode in the subcutaneous tissue of the upper arm. A random effects linear regression model was used to analyze the effect of intervention.

**Results:**

Overall, mean (± SD) PsqO_2_ was significantly higher in obese patients receiving goal-directed therapy compared to conventional fluid therapy (65.8 ± 28.0 mmHg vs. 53.7 ± 21.7, respectively; repeated measures design adjusted difference: 13.0 mmHg [95% CI 2.3 to 23.7; *p* = 0.017]). No effect was seen intraoperatively (69.6 ± 27.9 mmHg vs. 61.4 ± 28.8, difference: 9.7 mmHg [95% CI -3.8 to 23.2; *p* = 0.160]); however, goal-directed fluid management improved PsqO_2_ in the early postoperative phase (63.1 ± 27.9 mmHg vs. 48.4 ± 12.5, difference: 14.5 mmHg [95% CI 4.1 to 24.9; *p* = 0.006]). Intraoperative fluid requirements did not differ between the two groups.

**Conclusions:**

Goal-directed fluid therapy improved subcutaneous tissue oxygenation in obese patients. This effect was more pronounced in the early postoperative period.

**Clinical Trial Number and Registry:**

The study was registered at ClinicalTrials.gov (NCT 01052519).

## Introduction

Obesity is a major health care problem and its prevalence is rapidly increasing worldwide [1]. Laparoscopic bariatric surgery offers the most effective treatment to produce sustained weight loss and to resolve obesity-associated comorbidities [2]. Obesity is accompanied by significant pathophysiological conditions; therefore, obese patients require specific perioperative care [3]. Several guidelines and consensus statements for the anesthetic management of obese surgical patients have recently been developed [4, 5]. However, generally accepted evidence-based recommendations on fluid management are scarce [6]. Obesity is associated with body composition alterations mainly affecting the different fluid compartments. Absolute fluid volumes and thus cardiac output are increased [7], while the ratio of blood volume to total body weight decreases in a non-linear manner with an increase in body mass index (BMI) [8, 9]. Blood flow in the adipose tissue is inversely related to fat cell mass [10]. Thus, the estimation of fluid requirements to maintain normovolemia and hence optimized tissue perfusion and oxygenation remains challenging during the perioperative period. Inadequate tissue perfusion and hypoxia increase postoperative morbidity [11]. Subcutaneous tissue oxygenation is crucial for wound healing and serves as a major predictive factor for the development of surgical wound infections [12, 13]. In obese patients undergoing anesthesia and surgery, subcutaneous tissue oxygen tension (PsqO_2_) was substantially reduced compared to non-obese patients, and even supplemental oxygen failed to increase tissue oxygen availability [14, 15]. Beside factors such as the capillary rarefication of adipose tissue [16] and the increased sympathetic activity with subsequent alpha-adrenergic vasoconstriction [17], a suboptimal intravascular volume status might also have contributed to the observed tissue hypoxia. At present, individualized goal-directed fluid therapy (GDFT) is the most effective way to optimize cardiac performance and to improve oxygen delivery in the perioperative period [13]. We therefore evaluated perioperative subcutaneous tissue oxygenation in obese patients receiving GDFT compared to conventional fluid treatment. More specifically, we tested the hypothesis that intra- and postoperative PsqO_2_ is significantly reduced in obese patients undergoing conventional fluid management (control group) compared to goal-directed fluid administration.

## Methods

### Study Design and Objectives

This investigator-initiated, prospective, randomized, controlled trial was conducted at a single center (Medical University of Vienna, Austria). The study was approved by the ethics committee of the Medical University Vienna and was performed in compliance with the Good Clinical Practice guidelines and the principles of the Declaration of Helsinki. The study was registered at ClinicalTrials.gov (NCT 01052519) and reported in accordance with the recommendations in the CONSORT statement [18].

### Study Population

In total, 60 patients aged 18–65 years with a BMI ≥ 30 kg/m^2^ scheduled for laparoscopic gastric bypass surgery were recruited for this study. Exclusion criteria included decompensated heart failure, documented coronary or peripheral artery disease, renal insufficiency, severe chronic obstructive disease, and insulin-dependent diabetes mellitus. Furthermore, we did not include patients with obstructive sleep apnea syndrome requiring continuous positive airway pressure overnight. We also excluded patients with any known aortic or esophageal abnormalities, other than gastro-esophageal reflux.

### Randomization

After induction of general anesthesia and insertion of the esophageal probe for Doppler monitoring, obese patients were randomized into either the goal-directed obese group (GDFT) or the control group (CG) using a random number generator in sealed envelopes.

### Anesthetic Management

Prior to surgery, 500 mL of lactated Ringer’s solution (LR) was administered intravenously. Following a preoxygenation period of 5 min with 18 L oxygen flow, general anesthesia was induced with fentanyl (2–3 μg kg^−1^ ideal body weight), propofol (2–3 mg kg^−1^ total body weight), and rocuronium (0.9 mg kg^−1^ ideal body weight), and direct view laryngoscopy was performed in the ramped position. After induction of anesthesia, a 20G cannula was inserted into a radial artery. To standardize tissue oxygen availability in all patients, inspired oxygen fraction (FiO_2_) was adapted during the intraoperative period to maintain arterial oxygen pressure (PaO_2_) near 250 mmHg. Patients were mechanically ventilated with a tidal volume of 6–8 mL kg^−1^ ideal body weight at a rate sufficient to maintain end-tidal carbon dioxide partial pressure (etCO_2_) near 40 mmHg. Positive end-expiratory pressure was set between 7 and 10 cm H_2_O, and peak airway pressure was kept below 30 cm H_2_O. Subsequently, general anesthesia was maintained with sevoflurane in a carrier gas of inspired oxygen and air. A supplemental bolus dose of fentanyl (100 μg) was given when heart rate or arterial pressure exceeded 120% of the baseline value. Repeated doses of rocuronium were administered as deemed necessary.

Upper-body forced air warming (Bair Hugger system, 3M, MN) was used to keep patients normothermic, while local warming of the measurement site was strictly avoided. After induction, all patients were placed in the 25° reverse Trendelenburg position for the subsequent laparoscopic procedure. Capnoperitoneum was routinely maintained at 15 mmHg in obese patients. At the end of the procedure, following complete reversal of neuromuscular blockade, all patients were extubated in the semi recumbent position following manual hyperinflation and transferred to the postoperative anesthesia care unit (PACU).

### Intraoperative Fluid Management

After induction of anesthesia, a lubricated esophageal Doppler probe (CardioQ, Deltex Medical Group PLC, UK) was inserted orally into the esophagus and advanced to the mid-thoracic level to monitor stroke volume (SV) and corrected flow time (FTc). Measurements started as soon as an optimum descending aortic waveform was obtained and were continued during surgery whenever possible. During placement of the calibration bougie for the surgical procedure, the probe was retracted and Doppler monitoring was discontinued for approximately two measurements.

In the GDFT group, fluid management was strictly controlled. A maintenance rate of 2 mL kg^−1^ h^−1^ of crystalloid normalized to ideal body weight, which was calculated according to the Robinson formula [19], was administered throughout the entire study period. Additional fluid was administered based on a previously published and validated algorithm [20]. In short, patients received additional boluses of 250 mL LR guided by esophageal Doppler monitoring in order to achieve stroke volume optimization. Fluid responsiveness was defined as an increase in stroke volume > 10%. Boluses were given as long as fluid responsiveness was verified. If SV increased ≤ 10% after bolus administration, the patient was considered “non-responsive” and received another fluid bolus only when SV decreased > 10% from the most recent baseline.

The CG received fluid management according to the attending anesthesiologist. In this case, the esophageal Doppler monitor was turned away from the anesthesia care provider and the screen was covered with an opaque card. A researcher not involved in fluid management collected the displayed variables during surgery. In case of a mean arterial pressure < 65 mmHg and, as available, no Doppler-based signs of hypovolemia, intravenous vasopressors were administered at the discretion of the attending anesthesiologist.

### Postoperative Management

Patients were closely observed over 2 hours in the PACU after the surgical procedure. Postoperative pain was treated with intravenous fractionated piritramid and metamizol 1000 mg according to patients’ requirements by clinicians not involved in the study. Oxygen was delivered via a facemask to maintain PaO_2_ near 150 mmHg in the post-anesthesia care unit. In addition to the maintenance rate, crystalloid was given as deemed necessary by attending anesthesiologists.

### Measurements

Demographic and morphometric data, American Society of Anesthesiologists (ASA) physical status scores, comorbidities, and preoperative hemoglobin values were collected. We recorded routine variables, including duration of anesthesia and surgery, fluid balance data, hemodynamic- and Doppler-derived parameters, and anesthesia specific data. We measured intraoperative core temperature at the distal esophagus and forearm to fingertip skin temperature gradients with cutaneous Mon-a-Therm thermocouples as an indicator for arterio-venous shunt flow. Skin temperature gradients ≥ 0 °C were considered vasoconstriction [21]. Arterial blood gas analysis was performed as necessary to maintain PaO_2_ at the predetermined levels. Moreover, during their PACU stay, patients were asked to rate pain and nausea at 30-min intervals using a visual analog scale.

### Subcutaneous Tissue Oxygen Tension (PsqO_2_)

Our primary outcome parameter was intra- and postoperative subcutaneous tissue oxygen tension (PsqO_2_) measured from a surrogate wound in the upper arm. After induction of anesthesia, an implantable Silastic tonometer was inserted into the subcutaneous tissue of the right lateral upper arm for the measurement of PsqO_2_ and tissue temperature (TsqO_2_). Each tonometer consisted of 15 cm of tubing filled with hypoxic saline, with 10 cm of the tubing being tunneled subcutaneously. A Clark-type oxygen sensor and thermistor (Licox, Gesellschaft für Medizinische Sondensysteme, GmbH, Kiel, Germany) were inserted into the subcutaneous portion of both tonometers as previously described [22]. The disposable micro-probes were connected to a digital bedside monitor (Licox, Gesellschaft für Medizinische Sondensysteme, GmbH, Kiel, Germany), which displayed tissue oxygen and temperature values continuously. Recording of PsqO_2_ started after an equilibration time of 30 min and was continued during the intraoperative and postoperative study period. The probes were removed from the patient before transfer to the surgical ward.

In vitro accuracy of the oxygen sensors is ± 3 mmHg for the range from 0 to 100 mmHg, and ± 5% for 100 to 360 mmHg (in a water bath at 37 °C). Temperature sensitivity is 0.25%/°C, thermistors were incorporated into the probes, and temperature compensation was included in the PsqO_2_ calculations. Oxygen sensor calibration remains stable (within 8% of baseline value for room air) in vivo for at least 8 h. The electrodes are individually factory-calibrated, but calibration was confirmed by exposing the electrode to room air (ambient PO_2_ of 154 mmHg); in all cases, measurements in air were within 10% of 154 mmHg. To exclude a significant drift of the oxygen sensor, probes were again exposed to room air after each investigation; none differed by more than 10% from baseline values.

### Sample Size Consideration and Statistical Analysis

Sample size consideration was based on previous studies evaluating the effect of obesity on tissue oxygen tension [14, 15]. Based on the assumption of an average difference in PsqO_2_ of approximately 15 mmHg between the BMI groups, and an average standard deviation of 15 mmHg, 27 patients in each group would be required. This is based on the acceptance of a type I error risk of 5% and a type II error risk of 10% (power 90%). We therefore included 30 patients in each group.

We presented metric data as mean ± between subject standard deviation; not normally distributed data were presented as median and interquartile range (IQR); categorized variables are presented as absolute count and relative frequency. The data analysis follows the intention-to-treat principle. The primary outcome was the metric variable PsqO_2_, which we assumed to be normally distributed. To allow for a variable number of repeated measurements, we used random effects linear regression with treatment group allocation as the explaining covariable to estimate the difference in the outcome between the two groups. We present the effect of the intervention as the mean difference between the intervention group and control group together with a 95% confidence interval. We present separate estimates for the intraoperative phase as well as for the postoperative phase. A sensitivity analysis with the naturally log transformed PsqO_2_ as the outcome variable was performed to investigate the normality assumption. We analyzed metric secondary outcomes using ordinary linear regression or *t* tests with treatment group allocation as the explaining covariable. Binary secondary outcomes were analyzed using logistic regression models. For secondary outcomes with repeated measures, we used random effects regression as detailed above. To investigate randomization success, we tabulated baseline characteristics and compared these formally between the treatment groups. For metric variables, we used the independent sample *t* test or a Mann-Whitney *U* test; for categorized variables, we used the Fisher’s exact test. For data management and analysis, we used MS Excel and Stata 14.0 (Stata Corp, College Station, TX). Generally, a two-sided *p* value < 0.05 was considered statistically significant.

## Results

**Fig. 1:**
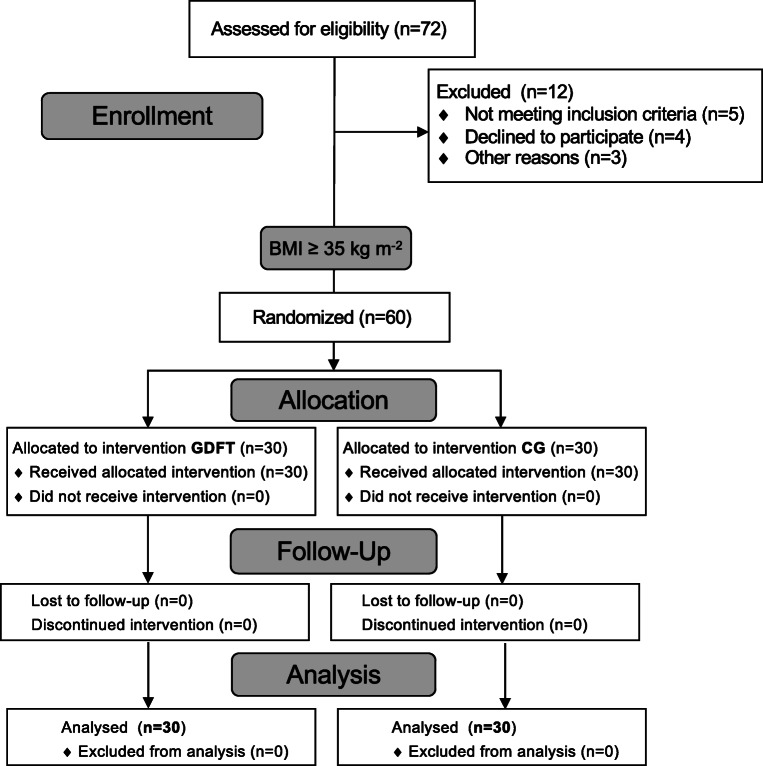
Flow diagram of participants included in the study according to CONSORT recommendation (GDFT: goal-directed group; CG: control group)

In total, 60 patients were included and randomized to receive either goal-directed or conventional fluid treatment (Fig. 1). All included patients received the allocated intervention according to the protocol; there were no cases in which the assigned treatment was discontinued. Demographic and baseline characteristics of included patients are shown in Table 1; there were no significant differences between the two groups.

**Table 1:** Demographic and baseline patient characteristics

Parameters	GDFT group (*n* = 30)	Control group (*n* = 30)	*P* value
Baseline data			
Age (years), mean (SD)	38 (11)	39 (11)	0.760
Female sex, *n* (%)	24 (80)	25 (83)	1.000
Weight (cm), mean (SD)	128 (17)	131 (21)	0.545
Height (kg), mean (SD)	168 (8)	167 (10)	0.817
BMI (kg m^−2^), mean (SD)	45.1 (3.9)	45.8 (6.3)	0.609
ASA score, *n* (%)			0.301
1	3 (10)	4 (13)	
2	24 (80)	19 (63)	
3	3 (10)	7 (23)	
Comorbidities			
Arterial hypertension, *n* (%)	7 (23)	13 (43)	0.170
NIDDM, *n* (%)	3 (10)	8 (27)	0.181
Smoker, *n* (%)	16 (53)	9 (30)	0.115
Hb preoperative (g dl^−1^), mean (SD)	13.5 (1.3)	13.6 (1.1)	0.628

Data are expressed as mean (SD), if not otherwise specified

*GDFT*, goal-directed fluid therapy; *SD*, standard deviation; *BMI*, body mass index; *ASA*, American Society of Anesthesiologists; *NIDDM*, non-insulin-dependent diabetes mellitus; *Hb*, hemoglobin

Continuous data in groups were compared using *t* test; discrete data were compared using chi-square test

### Subcutaneous Tissue Oxygen Tension

**Fig. 2:**
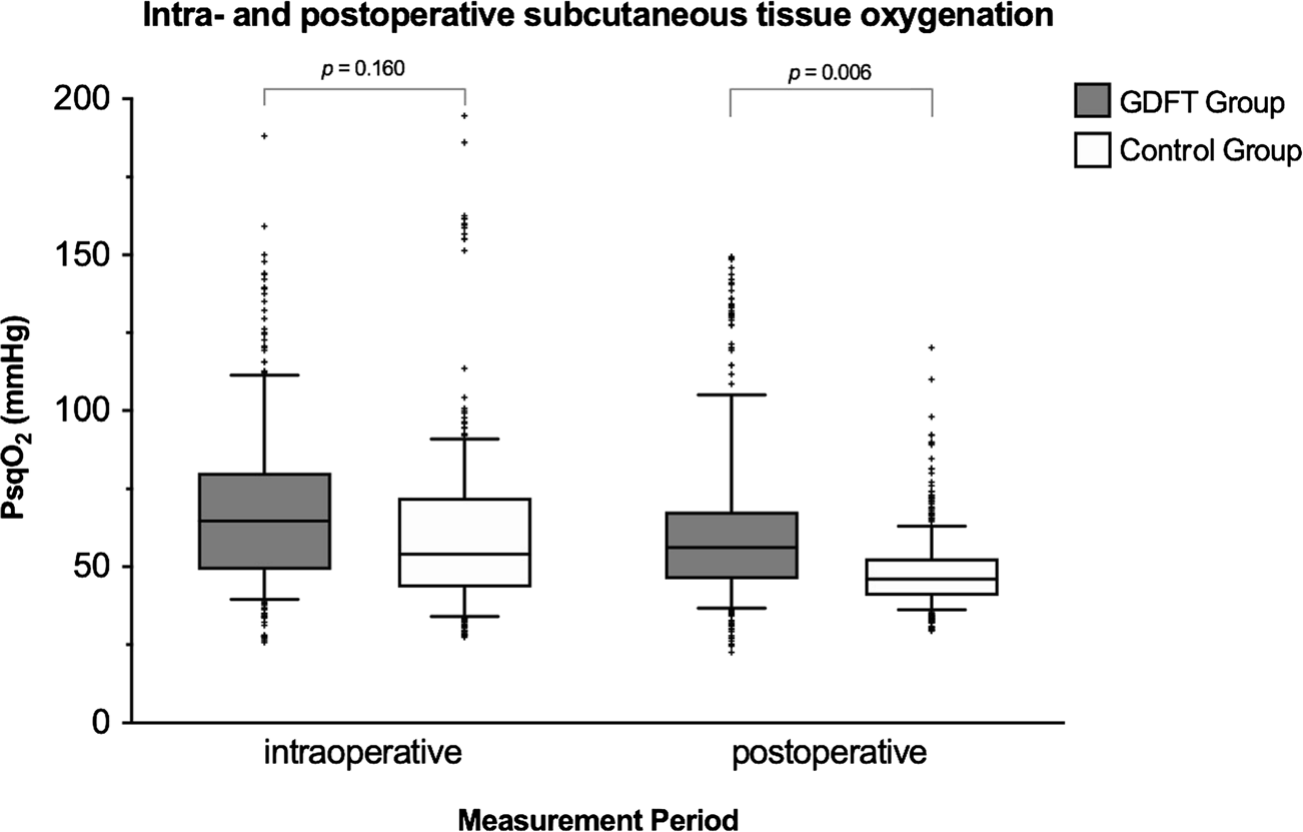
Intraoperative and postoperative subcutaneous tissue oxygen tension in patients undergoing laparoscopic gastric bypass surgery. Box plots indicate the median and interquartile range of PsqO_2_ values (mmHg) measured in the subcutaneous tissue of the arm (*upper arm*) in the two patient groups (median: bar; interquartile range: box; 10th to 90th percentile: whiskers; “+”: outliers); goal-directed group (GDFT group; *gray*) and conventional group (control group; *white*). For statistical comparisons, a random effects GLS regression model was used

Measurement of intra- and postoperative subcutaneous tissue oxygen tension was performed in all of the included patients. Overall, mean (± SD) PsqO_2_ was significantly higher in obese patients receiving goal-directed therapy compared to conventional fluid therapy (65.8 ± 28.0 mmHg vs. 53.7 ± 21.7, respectively; repeated measures design adjusted difference: 13.0 mmHg [95% CI 2.3 to 23.7; *p* = 0.017]). The intraoperative PsqO_2_ showed no significant difference (69.6 ± 27.9 mmHg vs. 61.4 ± 28.8, repeated measures design adjusted difference: 9.7 mmHg [95% CI -3.8 to 23.2; *p* = 0.160]). However, in the postoperative period, PsqO_2_ improved significantly in the GDFT group (63.1 ± 27.9 mmHg vs. 48.4 ± 12.5, repeated measures design adjusted difference: 14.5 mmHg [95% CI 4.1 to 24.9; *p* = 0.006]) (Fig. 2).

### Intraoperative Period

**Fig. 3:**
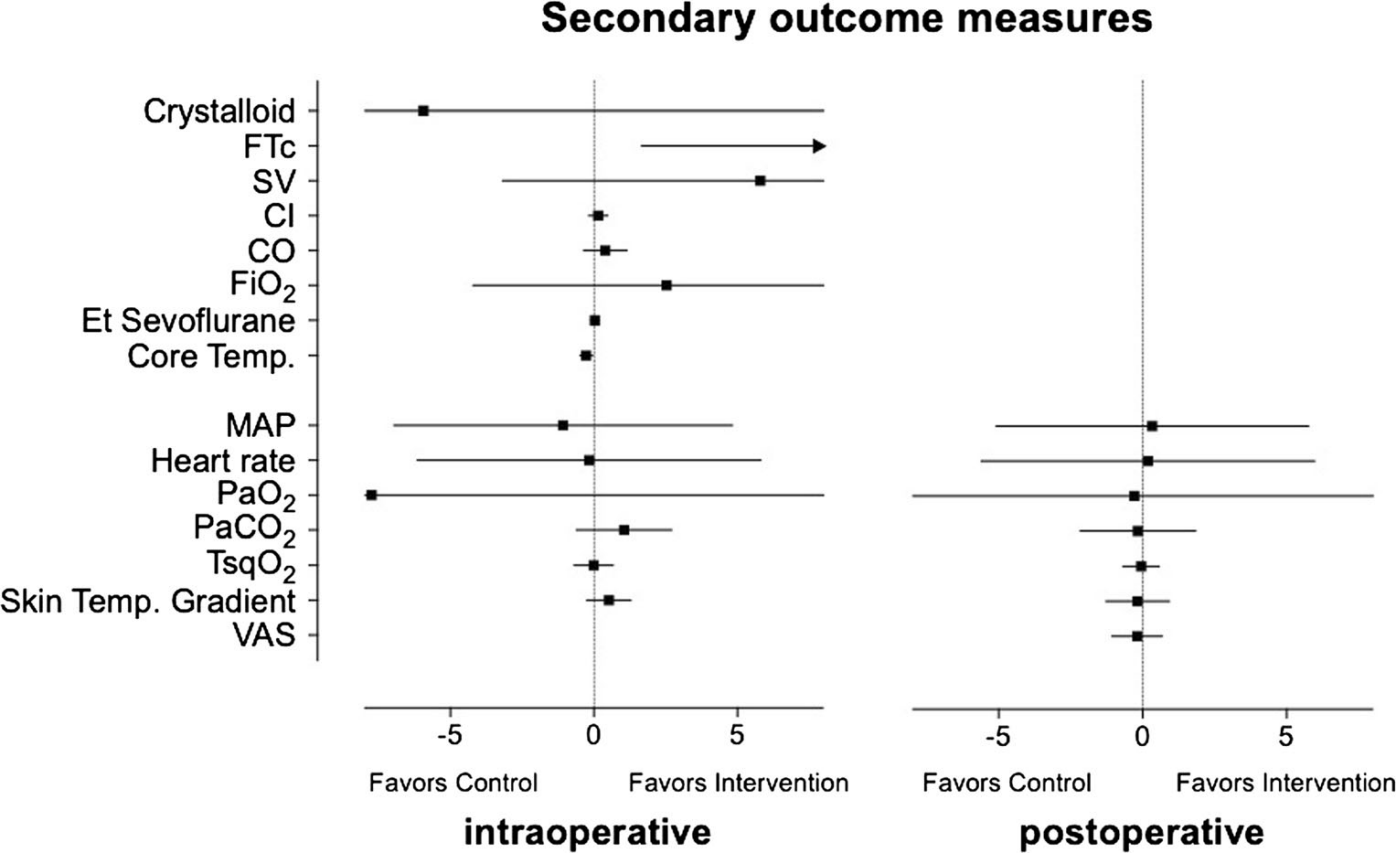
Secondary outcome measures. Forest plot showing interaction of potential modifiers on the main effect (coefficient: box or arrow indicating its direction; 95% confidence interval: error bars) for the intraoperative and the postoperative period. FTc, corrected flow time; SV, stroke volume; CI, cardiac index; CO, cardiac output; FiO_2_, inspired oxygen fraction; Et Sevoflurane, end-tidal sevoflurane concentration; Core Temp., core temperature at the distal esophagus; MAP, mean arterial pressure; TsqO_2_, subcutaneous tissue temperature; skin temp. gradient, forearm-fingertip skin temperature; VAS, visual analog scale

Duration of anesthesia and surgery was comparable between the groups. It is also noteworthy that fluid requirements did not differ between the two groups (for details, see Table 2). Anesthetic management was comparable between the groups. Patients assigned to goal-directed fluid therapy received a median dose of 0 mg (IQR 0 to 0.12) phenylephrine while patients in the control group received 0 mg (IQR 0 to 0.08) phenylephrine (*p* = 0.478) during surgery. Furthermore, there was no effect of vasopressor use on overall tissue oxygenation (coefficient: − 7.7 mmHg [95% CI − 34.1 to 18.7; *p* = 0.568]). As expected, mean (± SD) corrected flow time (FTc) was significantly higher in the goal-directed group compared to standard treatment (357 ± 33 msec vs. 339 ± 42, respectively; repeated measures design adjusted difference: 16 msec [95% CI 2 to 30; *p* = 0.029]). All other hemodynamics and Doppler-derived parameters (mean arterial pressure, heart rate, stroke volume, cardiac index, and cardiac output) did not differ between goal-directed and conventional fluid management in obese patients (Table 2, Fig. 3). Intraoperative core temperature was interestingly minimal different between the two groups (36.2 ± 0.5 °C vs. 36.5 ± 0.5, repeated measures design adjusted difference: − 0.3 °C [95% CI − 0.5 to − 0; *p* = 0.017]), while skin temperature gradients showed no significant difference (Table 2).

**Table 2:** Intraoperative management

Parameters	GDFT group (*n* = 30)	Control group (*n* = 30)	Estimate (95% CI)^†^
Duration			
Anesthesia (min), mean (SD)	156 (39)	162 (39)	6 (− 14 to 26)
Surgery (min), mean (SD)	110 (33)	106 (41)	− 4 (− 24 to 15)
Fluid management			
Crystalloid (mL), mean (SD)	1357 (393)	1363 (434)	− 6 (− 220 to 208)
Bolus (count), mean (SD)	4.3 (1.3)	0 (0)	4.3 (3.8–4.8)*
Hemodynamic and Doppler-derived parameters			
Mean arterial blood pressure (mmHg), mean (SD)	80 (15)	80 (14)	0 (− 5 to 6)
Heart rate (beats min^−1^), mean (SD)	75 (14)	75 (14)	0 (− 6 to 6)
Corrected flow time (msec), mean (SD)	357 (33)	339 (42)	16 (2 to 30)*
Stroke volume (mL), mean (SD)	81 (21)	74 (24)	6 (− 3 to 15)
Cardiac index (L min^−1^ m^−2^), mean (SD)	2.6 (0.7)	2.4 (0.9)	0.1 (− 0.2 to 0.5)
Cardiac output (L min^−1^), mean (SD)	5.9 (1.7)	5.4 (1.9)	0.4 (− 0.4 to 1.2)
Anesthetic management			
Fraction of inspired oxygen (vol%), mean (SD)	72 (15)	69 (15)	3 (− 4 to 9)
Et Sevoflurane (vol%), mean (SD)	2.0 (0.4)	2.0 (0.4)	0 (− 0.1 to 0.2)
Fentanyl (μg), mean (SD)	583159)	575 (230)	− 8 (− 111 to 94)
Phenylephrine (yes/no), *n* (%)	13 (43)	10 (33)	1.5 (0.5 to 4.4)^§^
Blood gas analysis and temperature			
PaO2 (mmHg), mean (SD)	229 (78)	236 (79)	− 8 (− 34 to 19)
PaCo2 (mmHg), mean (SD)	44.7 (4.8)	43.7 (5.2)	1.1 (− 0.6 to 2.7)
TsqO_2_ (°C), mean (SD)	32.5 (1.6)	32.5 (1.4)	0 (− 0.7 to 0.7)
Core temperature (°C), mean (SD)	36.2 (0.5)	36.5 (0.5)	− 0.3 (− 0.5 to 0)*
Skin temperature gradient (°C), mean (SD)	− 2.5 (1.8)	− 3.0 (1.8)	0.5 (− 0.2 to 1.3)

Data are expressed as mean (SD) or median, if not otherwise specified

*SD*, standard deviation; *PaO*_*2*_, arterial oxygen partial pressure; *PaCO*_*2*_, arterial carbon dioxide partial pressure; *TsqO*_*2*_, subcutaneous tissue temperature; *skin temperature gradient*, forearm-fingertip skin temperature; *CI*, confidence interval

For statistical comparisons, a random effects GLS regression model was used

^†^Difference between study groups if not otherwise defined

^§^Odds ratio

*p < 0.05

### Postoperative Period

During the postoperative period, no difference was seen regarding hemodynamic parameters (mean arterial pressure, heart rate) or temperature parameters (skin temperature gradient, TsqO_2_) (Table 3). None of our patients required vasopressor therapy during the postoperative period. However, intraoperative goal-directed fluid management improved PsqO_2_ in the postoperative period (63.1 ± 27.9 mmHg vs. 48.4 ± 12.5, repeated measures design adjusted difference: 14.5 mmHg [95% CI 4.1 to 24.9; *p* = 0.006]) (Fig. 2).

**Table 3:** Postoperative management

Parameters	GDFT group (*n* = 30)	Control group (*n* = 30)	Estimate (95% CI)^†^
Hemodynamic parameters			
Mean arterial blood pressure (mmHg), mean (SD)	107 (14)	108 (12)	− 1 (− 7 to 5)
Heart rate (beats min^−1^), mean (SD)	75 (12)	75 (13)	0 (− 6 to 6)
Blood gas analysis and temperature			
PaO_2_ (mmHg), mean (SD)	141 (44)	142 (45)	0 (− 18 to 17)
PaCo_2_ (mmHg), mean (SD)	42.4 (4.5)	42.2 (4.9)	0 (− 2.2 to 1.8)
TsqO_2_ (°C), mean (SD)	32.5 (1.5)	32.6 (1.6)	0 (− 0.6 to 0.6)
Skin temperature gradient (°C), mean (SD)	− 0.5 (2.6)	− 0.2 (2.5)	− 0.2 (− 1.3 to 0.9)
Postoperative course			
Urin (mL), mean (SD)	192 (207)	259 (245)	68 (− 49 to 185)
VAS, mean (SD)	3 (3)	3 (2)	0 (− 1 to 1)
PONV (yes/no), *n* (%)	19 (63)	19 (63)	0 (0 to 3)^§^
Antiemetic treatment (yes/no), *n* (%)	17 (57)	17 (57)	1 (0 to 3)^§^
Piritramid (mg), mean (SD)	9 (6)	8 (6)	− 1 (− 4 to 2)

Data are expressed as mean (SD) or median, if not otherwise specified

*SD*, standard deviation; *PaO*_*2*_, arterial oxygen partial pressure; *PaCO*_*2*_, arterial carbon dioxide partial pressure; *Tsq*, subcutaneous tissue temperature; *Skin temperature gradient*, forearm-fingertip skin temperature; *VAS*, visual analog scale; *PONV*, postoperative nausea and vomiting; *CI*, confidence interval

For statistical comparisons, a random effects GLS regression model was used

^†^Difference between study groups if not otherwise defined

^§^Odds ratio

*p < 0.05

## Discussion

Obesity, defined as a BMI ≥ 30 kg/m^2^, is a major determinant of perioperative tissue oxygenation. Subcutaneous tissue and wound hypoxia are common in obese patients during the perioperative period [14]. Even with supplemental oxygen, tissue oxygen tension is reduced to partial pressures, which are associated with a substantial increase in surgical site infections [12].

In this study, overall subcutaneous tissue oxygen tension increased significantly in obese patients undergoing laparoscopic surgery receiving Doppler-guided fluid administration. While no difference was observed intraoperatively, the effect of intervention resulted in raised tissue oxygenation in the early postoperative period. Our findings are in accordance with a recently published paper showing that subcutaneous tissue oxygen tension was high and well maintained in lean, overweight, and obese patients undergoing Doppler-guided fluid management during open gynecological surgery [23]. However, the previously published study did not include a representative control group and thus no direct comparison with patients receiving conventional fluid treatment was possible. This must be deemed a major limitation of this study. Additionally, obese patients had an average BMI of 33 kg/m^2^, which is far below the BMI range of the typical patient who is referred to weight loss surgery. The study presented here adds further weight to the evidence, as morbidly obese patients undergoing bariatric surgery with an average BMI of approximately 45 kg/m^2^ were included and randomly assigned to a treatment group and a conventional group.

We have previously shown that subcutaneous tissue oxygenation in obese patients undergoing laparoscopic surgery was reduced by roughly 15 mmHg compared to non-obese patients [15], which is comparable to our observed difference of 13 mmHg between both groups. However, in the abovementioned trial, patients received a fixed and rather low maintenance dose of fluid instead of an individualized guided fluid regimen. Thus, the absolute volumes administered were substantially lower when compared to our study. Our administered total volumes of fluid did not differ between the two groups. This is in accordance with recent evidence that BMI, at least in a lower range, does not determine fluid requirements [23]. There is evidence that the exact timing of bolus administration is a crucial factor and has more impact than the actual amount or type of fluid administered [13, 24, 25].

Laparoscopic case volumes have increased over time; especially in obese patients with an increased risk of wound healing complications, laparoscopic interventions represent state-of-the-art techniques. However, tissue oxygen tension is decreased during laparoscopic colonic surgery compared to open surgery [26]. Duration of surgery was considerably longer and arterial oxygen partial pressures were maintained at a lower level compared to our study. Thus, indirect comparisons between both studies are not feasible. During pneumoperitoneum, systemic vascular resistance is increased, which in turn decreases cardiac output [27].

Interestingly, our observed improvement of tissue oxygen tension in obese patients assigned to the goal-directed group was not reflected by systemic hemodynamics, which remained unchanged between both groups. One may argue that goal-directed volume optimization has a more pronounced effect during open abdominal procedures. So far, only a few studies have focused on the coherence between optimized global hemodynamics and microcirculation during perioperative GDFT [28, 29]. In a heterogeneous intensive care population, microvascular flow index improved independently of changes in stroke volume after fluid challenge [30]. This indicates that timely fluid administration enhances impaired microcirculation, i.e., peripheral blood flow and thus oxygen delivery to peripheral tissues independently of microcirculation.

Given the cardiovascular peculiarities of obese patients, the assumption of hemodynamic coherence, in the specific setting of pneumoperitoneum, remains questionable. Alongside macro- and microcirculation, arterial oxygenation constitutes a major determinate of tissue oxygen availability [31]. One strength of our study is that intraoperative arterial partial pressures were comparable and well controlled in both groups. The benefit of intraoperative individualized GDFT in terms of postoperative morbidity is well established [32]. So far, only a few studies have evaluated GDFT protocols in bariatric patients. In general, treatment algorithms based on dynamic preload parameters such as stroke volume variation or pleth variability index and their effect on fluid requirements and postoperative renal function have been evaluated [33, 34]. Stroke volume optimization according to arterial pressure waveform analysis decreased the incidence of postoperative nausea and vomiting and shortened length of hospital stay [35]. As far as we are aware, we have shown for the first time the feasibility of esophageal Doppler monitoring in this specific setting; except for approximately two measurements during placement of the calibration bougie for gastric pouch sizing, we were able to obtain stable Doppler signals over the time period. Compared to dynamic preload parameters, esophageal Doppler monitoring is not influenced by tidal volume and increased abdominal pressure, and might therefore provide more reliability during the specific condition of pneumoperitoneum.

The use of vasopressors and subsequent vasoconstriction is an important confounding factor for tissue oxygen tension. However, phenylephrine use in our study was comparable in both arms and had no effect on tissue oxygen tension.

A limitation is that we did not measure gut or wound tissue oxygen tension but instead evaluated PsqO_2_ from a needle-induced surrogate wound in the upper arm. This represents the classical method of measuring tissue oxygenation and has been validated and used in numerous previous studies [22]. The benefit of this location is that measurements can be conducted easily during surgery. Microscopic tissue injury to subcutaneous tissues at the sensor insertion site in the upper arm is not only inevitable, but is indeed a deliberate feature of this model, which is designed to mimic surgical trauma [36].

All our patients were relatively healthy; obese patients were predominately female and procedures were short. The beneficial effect of guided stroke volume optimization might be more obvious in patients with cardiovascular compromise, in male patients with a higher degree of central obesity and during longer procedures with more extended fluid shifts. We did not expect any long-lasting effects of our fluid regimen, which was restricted to the intraoperative period. Thus, we only measured tissue oxygen tension during a short period of time in the immediate postoperative period. The first hours after surgical trauma are considered to represent a decisive period for the establishment of surgical site infection and are thus of major interest [37].

## Conclusion

Regardless of similar systemic hemodynamics and comparable fluid requirements, goal-directed fluid therapy improved subcutaneous tissue oxygen partial pressure in the early postoperative period in obese patients undergoing laparoscopic bariatric surgery. To enhance peripheral perfusion and thus tissue oxygen availability, the timely administration of fluid boluses according to patients’ needs might be more important than the total amount of fluid. Given the laparoscopic approach and the short duration of surgery, our findings might have more impact in patients undergoing open abdominal surgery associated with significant fluid shifts.
